# Understanding support needs of African and African-Caribbean people living with dementia, their care partners and families, and impacts of delayed support: Identifying inclusive strategies to facilitate social care support: A study protocol

**DOI:** 10.1371/journal.pone.0354661

**Published:** 2026-07-27

**Authors:** Shadreck Mwale, Andy Northcott, Brenda Hayanga, Deborah Edwards, David Truswell, Catherine Coveney, Juanita Hoe, Megan Wyatt, Shabana Roscoe, Katie Featherstone

**Affiliations:** 1 The Geller Institute of Ageing and Memory, University of West London, London, United Kingdom; 2 Global Health and Social Medicine, Kings College London, London, United Kingdom; 3 JBI Wales Centre For Evidence Based Care, Cardiff University, Cardiff, United Kingdom; 4 PPI Representative; 5 School of Social Sciences and Humanities, University of Loughborough, Loughborough, United Kingdom; PLOS: Public Library of Science, UNITED KINGDOM OF GREAT BRITAIN AND NORTHERN IRELAND

## Abstract

African and African-Caribbean (AAC) people living with dementia (PLWD) are a population at high risk of inequitable access to health and social care services, and have poor health and wellbeing outcomes. Research suggests they are likely to experience failings in care, be recognised by services late and at points of crisis, and are at increased risk of institutionalisation. This research aims to examine the experiences of AAC PLWD, their care partners and families of accessing social care services and support. Employing an intersectionality theoretical framework, this study will use a multi-method, flexible exploratory sequential design. An evidence synthesis using the JBI meta-aggregation approach, co-produced with AAC PLWD, their care partners, and families, will be conducted to synthesise existing evidence on the experiences of AAC PLWD, their care partners’ and families’ of support seeking in the UK. Narrative interviews will involve 3 sequential interviews conducted with 40 AAC PLWD (n = 10 per site), with opportunities for dyadic interviews with care partners and families. Ethnographic fieldwork will be conducted across 4 Local Authority sites within Adult Social Care teams (n = 30 days per site) to provide insight into institutional and organisational processes and cultures, and staff practices and engagement with PLWD. Artistic art workshops will be conducted to facilitate a diverse range of participant voices and support meaningful engagement for AAC PLWD at increased risk of isolation. Ethical approval has been obtained. Dissemination will include peer reviewed publications, conference presentations and free publicly available resources for health and social care professionals, third-sector partners, PLWD and their carer partners.

## Introduction

African and African-Caribbean (AAC) people living with dementia (PLWD) are a key population at high risk of inequitable access to and adverse experiences of health and social care services [1,2], experience failings in care, and have poor health and wellbeing outcomes [3–5]. This pattern of inequalities has been identified for over 20 years [4,5]. Our preliminary systematic scoping search identified only 9 studies published during this period that have focussed on or included PLWD from AAC backgrounds living in the UK. These have focused on a wide range of topics including service use [6,7], dementia severity at presentation to memory services [2,8], screening [9], barriers to help seeking [10] awareness raising [11], risks [12], and prevalence of dementia [1] compared to White British PLWD. Research has yet to address the needs of older AAC PLWD, examine their experiences and the impacts of delayed recognition by health and social care services, or provide the evidence required to support ‘timely’ access to services or access to services once they have reached crisis [1,2,10,13].

By African and African-Caribbean we refer to people whose heritage broadly originate from Africa and Caribbean countries (formerly known as ‘The West Indies’), and other ‘Caribbean Community and Common Market’ (CARICOM) territories [14]. There are no official figures identifying the population of PLWD from AAC communities in the UK, within the approximately 25,000 PLWD from minoritised ethnic communities [11] – a population projected to increase seven-fold by 2051 [2].

AAC PLWD face significant challenges accessing services early and timely [15–18]. They are the population most likely to be recognised by and to reach services late [1,2,10,19] and are less likely to seek support from formal health and social care services [19,20]. Late recognition [21,22] and support by services, and delayed support seeking by AAC PLWD [1,2] means they are identified by services only once they have reached a more advanced stage of their dementia [1,2], have reached the point of crisis [1,2,10,11] and require urgent acute medical and social care intervention.

These patterns of potentially avoidable acute hospital admission at crisis can further perpetuate inequalities following discharge, with AAC PLWD [23] at high risk of being missed by services [17], and a premature move into long-term care [24–27]. Misrecognition of a person’s needs [21] by health and social care services, and delays in support seeking [24,28,29] mean AAC PLWD are also more likely than other populations to experience ‘crisis’ [10,19,20].

A crisis is characterised by a sudden episode resulting in the perceived inability of individuals to cope [30,31], draw on support, live independently [24,28,31], and a possible refusal or delay in seeking mainstream social care support [29] until the PLWD requires urgent acute medical and social care intervention and increased and intensive support. Once a person experiences crisis, this is associated with significant physical and cognitive deterioration [19,28,30–32] poor health and wellbeing outcomes [32–35], and unplanned acute hospital admissions [36,37]. Older people who experience acute unscheduled hospitalisation are at increased risk of further physical and cognitive decline [38]. Carer burden, which is usually preceded by crisis, is the most significant factor contributing to the institutionalisation of a PLWD [27,39].

### Knowledge gap

The limited research examining the experiences of AAC PLWD is in part because research strategies have habitually grouped diverse minoritised ethnic communities together [13,19,40]; AAC and people from South Asian communities [20,41]; AAC, South Asian, and White British communities [42,43]; AAC, South Asian and Chinese communities [44]; AAC and Greek communities [45]; or AAC populations as providing a comparative examination with White British PLWD [1,2,12,46]. In addition, studies have typically aggregated diverse minoritised ethnic communities together (including AAC PLWD), to examine carers’ experiences; support seeking [2], stigma [47], racism [47], institutionalisation [12] and equality of service provision [48].

This established research approach has resulted in homogenous portrayals of minoritised ethnic communities experiences, which fail to recognise difference and diversity [15,17,18,40,49–51]. Unsurprisingly, this approach has resulted in this body of research reinforcing stereotypical narratives, through delivering a body of findings which focus on highlighting perceived failings within the community, including religious and cultural beliefs [16,17,19] knowledge deficit [19,51,52], and cultural perceptions of illness [10,52] as explanations for late recognition by services and for support seeking at crisis.

### Aims and objectives

The aim of this three-year (2025–2027) National Institute for Health and Care Research (Award ID: NIHR160824) funded study is to examine the everyday practices and visible work of Adult Social Care teams when recognising, assessing, responding to and supporting the needs of AAC PLWD. It will explore how staff understand and make decisions about care and how these practices shape the experiences of PLWD, their care partners, and families. The study will also investigate how AAC communities navigate support-seeking and engage with Adult Social Care services.

The research addresses two key questions:

1. How do Adult Social Care teams understand, recognise, assess and respond to the needs of AAC PLWD?
2. What are the experiences of support seeking from the perspectives of PLWD from AAC communities, their care partners, and families living in the UK?

The objectives are to:

1. Develop a detailed conceptual model of help-seeking to explain what is known of pathways and access, and experiences of services that supports the provision of culturally appropriate care for AAC PLWD.
2. Examine and understand the experiences of AAC PLWD, care partners and families of support seeking and encounters with services.
3. Provide detailed understandings and directly observed examples of recognition, assessment, and support of AAC PLWD, care partners and families, by Adult Social Care teams.
4. Translate our findings into co-produced culturally appropriate and inclusive strategies that are actionable.

## Materials and methods

This multi methods study uses a range of perspectives and methods – the “building blocks of evidence” – to produce new knowledge [53]. We utilise a flexible exploratory sequential design integrating an evidence synthesis (co-produced evidence synthesis meta-aggregating findings) and qualitative methods incorporating case studies (narrative interviews), ethnography (observations and short in-situ ethnographic interviews, documentary analysis), and co-production (translation). This approach of structured discovery utilises focussed methods, which allows flexibility to capture unexpected findings and key social processes [54].

### Intersectional theoretical framework

Our theoretical approach recognises social categories intersect to produce varied and multiple health inequities, which reflects an important shift away from viewing the social determinants of health and health inequities through a single-axis framework [55,56]. In response, we utilise intersectionality to provide a theoretical framework to examine how intersecting power relations across multiple and layered identity positions, including race, gender, class, ability, ethnicity, age, citizenship, sexual orientation, and nation, influence social relations [57].

Intersectionality, a term conined by Kimberle Crenshaw in 1989, originates from Black feminist thought, which seeks to challenge the idea that there can be a universal understanding and experience of inequality, marginalisation, and subjugation. Crenshaw [58] illuminated the limitations of the single-axis frameworks which have a tendency to treat social categories as mutually exclusive categories of analysis and experience. Sojourner Truth’s famous speech at a Women’s Rights Convention in Ohio, USA in 1851 [59] is one of the earliest documented examples of intersectionality-informed interpretations of lived experience. Truth, a freed slave, argued that being a woman (gender) and Black (race) were not mutually exclusive, rather intersect to further subjugate Black women [59]. Contemporary feminism has built on this view, applying the idea in multiple academic disciplines to illustrate ways in which life experiences are impacted by multiple, layered, forms of discrimination, some of which may be overt and others more hidden or discreet. This expansive body of work explicates how Black women are marginalised and face oppression first as women and then due to their ethnicity [60,61]. Adding other factors such as sexual orientation and disability further adds to their marginalisation, subordination, and erasure [57,62].

This theoretical framework informs our focus on integrating diversity of experience into the design and conduct of our research to ensure inclusivity and to focus on the needs and experiences of vulnerable and marginalised groups who have been underrepresented thus far [63–65]. Analytically, adopting an intersectional framework enables us to bring together the subjective experiences of care with the wider social forces that act to shape these services [56,64,66–68].

Although a small number of reviews have applied an intersectional framework to dementia [63,69,70], no primary research has examined the ways in which PLWD’s unique, intersecting identities and social location, interact to shape their care experiences and access to support services. As a conceptual tool, intersectionality offers a way for us to examine the experiences of AAC PLWD, and to understand and illustrate how forms of subjugation exist for racialised vulnerable adults within care spaces. Taking an intersectional vantage point brings into view multiple axis of privilege and interlocking systems of oppression and marginalisation, including racism, classism, sexism, ageism and disablism that permeate these spaces and shape experiences of dementia care [71]. Moreover, it enables us to recognise the similarities and differences in conceptions of health and illness across and within the study populations, that shape individuals’ meanings and experiences of dementia and dementia care, including their illness narratives and support seeking behaviours.

#### Intersectional methodological approach.

An intersectional framework is embedded across all stages of this research, to ensure it acknowledges and accounts for the specific needs of the research populations, informing our approaches to recruitment, sampling, processes of taking informed consent, data collection methods, analysis, co-production, dissemination of findings, and pathways to impact.

Methodologically, a feminist intersectional approach means this research is grounded on the understandings that knowledge is situated, subjective, and derived from close interactions between the researcher and the subject of research [72–74]. Rather than seeing reality as objective and universal, feminist approaches draw attention to the subjective and contextual nature of knowledge and everyday social life [72,73]. We draw on a feminist intersectional approach which Star [74] describes as essentially a method for understanding perspectives from the margins or ‘boundary-dwelling perspectives’ [72,74]. This requires the research team to continually ask and search for what else may be going on or invisible in a social problem that may add to its complexity or further burden the affected groups, while staying attuned to the ways in which power inequality manifests [75]. This informs our approach to exploring organisational and societal cultures by examining the intersections of location and power relations in shaping knowledge and its impact on organisational practice and cultures, and everyday social life [60].

A significant strength of employing intersectionality as our methodological approach is that it helps detect the inescapable blind spots. As a result of our own experiences, social status, and identity, researchers can easily become oblivious to what our research data and materials offer. Such blind spots can undermine “even our best intentions to do research which is comprehensive and critical” [75]. Utilising an intersectional approach provides a lens through which we will re-examine taken for granted everyday experiences, assumptions, and outcomes to examine other vectors that may be contributing to the issues under investigation [75].

### Study design

The study will adopt a mixed methods approach, exploratory sequential design [76] integrating an evidence synthesis (a co-produced qualitative systematic review) and qualitative methods incorporating case studies (narrative interviews), ethnography (observations and short in- situ ethnographic interviews, documentary analysis), and translation (co-production). Using a structured discovery approach allows for the necessary flexibility to capture unexpected findings, key social processes and the generation of detailed contextual data to inform improvements in health care service delivery.

#### Evidence synthesis (Objective 1).

We will carry out a qualitative systematic review using the meta-aggregation approach to synthesise findings [77], whereby we will extract findings and illustrations that are specific to PLWD from AAC communities from studies and bring these together in a series of synthesised findings. Unlike other qualitative evidence synthesis approaches, meta-aggregation develops’recommendations for action‘by enabling the creation of generalisable statements [78]. An a priori protocol has been developed and registered with the international Prospective Register of Systematic Reviews [79] and reported following PRISMA guidance [80].

To ensure the review focus is relevant to the needs of AAC PLWD, and to the context of the social care and community settings, we will present and discuss the initial synthesised findings with stakeholders, who will include AAC PLWD, care partners, family members, and practitioners in social care and community third sector settings.

### Sampling and recruitment

Sampling in ethnographic research requires a flexible constant comparative approach in which sampling, data collection, and analysis occur iteratively and remain responsive to emerging insights. This allows attention to intersecting factors including class, gender, and social economic status among others [11,62]. Theoretical sampling offers a pragmatic approach, with sites, participants and sample size purposively based on variables likely to contour the phenomena. The flexible constant comparative approach allows comparisons within and across sites [81], strengthens the generalisability of findings [82] and their potential to inform practice and policy [83]. Adult Social Care settings are suited to an ethnographic approach. At first glance, Adult Social Care services, and the teams within them, may appear similar and homogenous; however, they all have unique cultures of working, contoured by local resources, cultures and organisational systems which in turn influence variance in care and decision making across and within institutions. We have Local Authorities (LAs) in Southeast England (two inner city and one coastal) and the Northwest of England (one encompassing both inner-city and coastal areas) to ensure variation in geographical and social economic status.

#### Sampling and recruitment for narrative interviews with African and African-Caribbean people living with dementia and their families (Objective 2).

We will use purposive and snowball sampling to include AAC PLWD from diverse socio-demographic backgrounds, including age, gender, socio-economic status, and groups within the communities who might be experiencing intersecting marginalisation as they are often underrepresented in research and/or missed by services. Participants will be recruited via community partners and champions. In collaboration with community partners, prior to recruitment we will deliver focused outreach activities in the community to sensitise communities about our research.

The participants for the narrative interview will be AAC PLWD. Participants will be 50 years of age or older, enabling the inclusion of participants living with young-onset dementia; and may or may not be currently accessing, or are yet to access, Adult Social Care support. We recognise that formal diagnoses of dementia are not always straightforward to establish. Therefore, we will include people who may be exploring a diagnosis or who may be suspected of having cognitive impairment.

Theoretical sampling will be used to include AAC PLWD across a wide range of socio-demographic characteristics. Using maximum variation, purposive, and snowball sampling to reflect diversity in age, nationality, immigration status, disability, sex and gender, socio-economic status, sexuality, spoken language, and religion. We will also capture variation in migration histories including ‘Windrush’ generation families and asylum seekers. Including voices from these underrepresented groups is important for developing robust, inclusive and culturally responsive policy and practice.

#### Sampling and recruitment for ethnographic component (Objective 3).

Across each LA Adult Social Care service, data collection will follow a standardised chronological approach. Theoretical sampling, informed by grounded theory, will be used to ensure that representativeness and consistency of concepts and events is achieved. The focus will be on ‘discovery’ to ensure the grounding of emerging concepts within data and the reality of the settings [84].

We will follow the everyday work of social workers and allied health professionals using purposive sampling to include a range of professional roles and grades, including duty social workers, occupational therapists, support workers and staff coordinating responsibilities. Several months before fieldwork begins, the research team will visit each Adult Social Care team to discuss the study’s aims and approach.

Staff working in Adult Social Care teams in the research sites, whose roles involve responding, assessing, organising and instituting social care for older PLWD (e.g., social worker, assistant social workers, occupation therapists, support workers) will be able to participate in the ethnographic component. During observations of staff members’ home visits, the focus will be on their practice and not the residents – we will only observe staff assessments and decisions. After each visit, we will have a short debrief (in-situ interview) with them to obtain insight into their decisions.

### Data collection

We will adopt a qualitative approach including ethnographic observations and in-depth qualitative interviews.

#### In-depth narrative interviews (Objective 2).

We will be conducting interviews with AAC PLWD and their families. We will utilise case studies using a narrative interview approach to allow for personal accounts, experiences, and stories to emerge and to provide depth and sensitivity of insights and understandings [85]. We will conduct interviews in a sequence of meetings over time (n = 3 with each individual), during which participants will be encouraged to reflect on the meanings, affect and thoughts about accessing services [86]. This approach will enable themes to emerge over time, ideas to be revisited, and reflections incorporated into individual and wider narratives across the cohort. This will enable mapping rationales and trajectories, including odysseys into accessing social care support, experiences of receiving and being supported by Adult Social Care teams, and impact of these experiences on health and wellbeing. Participants will be allowed time to direct and share their story over a number of visits; this was identified by our Research Advisory Group as an essential approach to support the involvement of PLWD.

Within each LA catchment area over a four-month period (with a further two-month follow up period) we will carry out narrative interviews with AAC PLWD (n = 10 per site, n = 40 total), who will take part in (n = 3) sequential interviews (n = 120 total), each lasting up to an hour. Interviews will be conducted over a four-month period (where possible care partners and families will also be involved). A further period of two months will allow for follow-up interviews with participants who were recruited at later stages within the site

Interviews will explore their dementia diagnosis odysseys; the rationales for support sought, trajectories for support seeking, networks and adjustments of support, care experiences, and the impacts of these experiences on their wellbeing. As part of the narrative approach, interviews will be accompanied by detailed field notes to provide details of context of each interview and will support the case study approach.

#### Artistic postal dialogue and art workshops (Objective 2).

To ensure diversity of participants voices in the study, we will conduct a series of art workshops for PLWD hosted within local community services across all (n = 4) sites. These creative art workshops will involve creatively engaging in a range of activities and with different art materials. Using the creative art workshops approach, we will support communication and expression of experiences for all participants including people living with advanced or rare dementias. If appropriate, during the workshops, participants will be invited to talk through their art and what it means to the subject of the research. The goal will be to ensure diverse voices are heard and represented in the study, as well as building trust and community participation in research.

To support the inclusion and involvement of AAC PLWD for whom verbal communication may be difficult, we will use participatory, visual, and textual creative methods and approaches, which are in keeping with our wider intersectional approach. We will invite those unable to participate in face-to-face workshops, interviews or further stages of interviews, to participate in artistic postal dialogues. This will involve PLWD (their care partners and families) forming a creative dialogue with an artist researcher, communicating with each other through developing artworks and sending and responding via post to build a dialogue together over time. The researcher, who is an experienced artist, will create an artwork in response to participants’ artworks and post this back to them. The process will then be repeated over the course of the study, with the end point directed by the participants.

#### Ethnography in Adult Social Care services (Objective 3).

A significant part of Institutional ethnography is examining the everyday work of people, their routine behaviour and practices and their articulation work, and how they account for and make sense of their actions [87]. An important focus is the frequently understudied, often characterised as the mundane and every day. Within any organisation there are always groups’ whose everyday work is not recognised formally and is often unnoticed and invisible [88].

We will focus observation on the largely invisible everyday work of Adult Social Care professionals. We will provide a detailed understanding of the social and institutional forces that shape and influence their work. Our ethnographic approach will enable us to understand how staff respond to the care needs of AAC PLWD and to follow the impacts of their actions on PLWD, their care partners and families. Importantly, we will also examine how they account for and make sense of their responses to care needs in these contexts. Ethnography allows us to examine these elements and, importantly, the interplay between them [89].

Fieldwork will be conducted in (n = 4) LA areas across England which are known to have large numbers of AAC people. Within each site we will carry out ethnographic data collection (observation, in-situ interviews, and documentary analysis) with (a) Adult Social Care teams (n = 30 days/shifts; n = 120 days total), (b) shadowing social care professionals (n = 5–10) and the team and multi-disciplinary meetings (MDT) they attend. In total, we will carry out ethnographic data collection within n = 12 teams.

We will:

- carry out focussed observations of team and case meetings, and the multi-disciplinary team meetings they attend, whose referrals and cases will include AAC PLWD
- shadow individual professionals who review and triage referrals and cases
- conduct in-situ short ethnographic interviews with staff following meetings and during shifts to explore rationales for actions and decisions (<5 minutes), and to obtain staff perspectives
- carry out documentary analysis (case files, team records including handover and case lists) of staffing, workload and case allocation, client acuity and placements, to provide context.

Across the Adult Social Care teams, we will include observations:

- shadowing the duty social worker as they respond to calls from the community to understand referrals
- shadowing social workers while on home visits to their clients to observe how needs and risks for clients are recognised, assessed, the rationales for decisions, and how support options are presented, negotiated, and agreed on. This will help us understand the visible work of social workers and the challenges they face in practice
- attending ‘Team around the client meetings’ and ‘Strategy Safeguarding Meetings’ where teams respond to urgent safeguarding concerns requiring immediate action or when safeguarding and similar issue arise.

In keeping with our intersectional approach, we will also carry out interviews with other professionals, such as occupational therapists, community nurses, mental health crisis teams, General Practitioners, and police involved in the care of PLWD, should they be involved in these meetings.

### Data analysis

Data collection and analysis will be informed by the analytic tradition of grounded theory [84] using the constant comparative method to ensure that insights from one site inform the focus of subsequent data collection and analysis. Field notes documenting observations, experiences, and near verbatim accounts will be written up promptly. All audio recordings of in-depth narrative interviews will be professionally transcribed verbatim to support rigorous analysis. Informed by an intersectionality framework, analysis will involve the development and testing of analytic concepts and categories through careful reading of the data, identifying patterns, relationships, inconsistencies, and unexpected insights across the range of perspectives gathered. To support this approach, we will employ an abductive analytical approach, synthesising empirical data with existing concepts, while being open to the emergence of new concepts [89]. The interplay between data, theory and emerging concepts supports our exploratory grounded interpretation that remains closely aligned with participants’ experiences and the organisational context in which these occur.

Analysis of interviews will involve examining and interpreting content of speech story by focusing on meaning, with the goal to scrutinise, reflect and theoretically interpret elements of the participants’ lived experiences in relation to support seeking [90,91]. Preliminary analysis of narrative interviews will keep individual accounts whole, bringing them together as individual case studies to deliver detailed accounts of the individual, their care partners and families, and their encounters with services [92]. A thematic approach will then be taken across the case studies by finding common thematic elements across research participants and the events they report. Once a typology is developed, the data will be reviewed and further refined to ensure accuracy of the accounts across the cases. Conversations from artistic workshops will not be analysed, but both notes and images will facilitate a deeper understanding of participants’ experiences.

Coding of ethnographic fieldnotes (observations and in-situ interviews) will produce analytic memos and a collection of sensitising concepts and ethnographic coding of decision-making events and scenarios. The abductive method means that the coding of data into categories will be an ongoing and recurring process, with further data then examined in the context of previous fieldwork and the analytic memos generated, then informing data collection at the next site [93].

The analysis of the narrative interviews and ethnographic observational data will be triangulated to form a master analytic report for each site. We will draw data from the evidence synthesis, narrative interviews and ethnographic observations to generate a detailed understanding of participants’ experiences of support seeking in social care. We will explicitly use intersectional perspectives to inform the development of our analysis to identify local organisational and structural conditions and how these contour access to support. Data will be managed using NVivo 15. This will also facilitate secure access to data for the wider research team.

### Patient and public involvement

The focus of this study was identified as a research priority by our Patient and Public Involvement (PPI) advisory group (referred to as Research Advisory Group), who also co-produced this proposal. These AAC PLWD reported feeling anger at being ignored, and reported a lack of understanding of their needs. This Research Advisory Group comprises ten people with lived experience – specifically: PLWD (n = 2), carers (n = 5), and managers of third sector organisations (n = 3) supporting PLWD. One of the co-applicant’s also has lived experience as carer of a family member with dementia. The Research Advisory Group was formed from long-standing community outreach across England, supported by a series of engagement and consultation events designed to build relationships and trust. The group will play a key part in this study. They will contribute to the development and drafting of the ethics applications, and the writing of the public facing documents, such as consent forms and participant information sheets. The Research Advisory Group and research team will meet monthly (in person and virtually), to support and provide advice to the researchers as the study progresses.

### Ethics

Ethical approval has been obtained from the West Midlands – Coventry & Warwickshire Research Ethics Committee and the Health Research Authority (CAG ref, 25/CAG/0058; REC Ref, 25/WM/0094), the Association of Directors of Adult Social Services (ADASS), and the School of Medicine and Biosciences, University of West London Research Ethics Panel (UWL/REC/SBS-01173). Consent will be obtained from all participants, and capacity will be assessed in keeping with Mental capacity Act (2005).

For the ethnographic component, prior to commencing observations, verbal consent will be obtained from the staff; this will be followed by written consent at a convenient time to ensure minimal disruption to their work. We will obtain verbal consent from staff at the time of interviews, to check if they are able to share their reflections on their practice with us. Before each home visit, staff will seek consent from those they will be visiting, letting them know the focus will be on their practice only. All staff will have provided written informed consent to be shadowed.

Written consent will be received from all participants before the narrative interviews take place. Verbal consent will be received before each interview takes place, with the experienced researchers also paying close attention to any physical or other signs throughout. If it is determined a participant appears to be losing capacity, losing interest or uncomfortable, the interview will be stopped immediately.

All narrative interview recordings will be professionally transcribed verbatim; field notes from observations and verbatim text of in-situ interviews will be written up into Microsoft Word files. Transcripts will be checked for accuracy and fully anonymised in line with Caldicott Principles.

### Dissemination

A combination of academic and publicly accessible outputs will be developed to support Adult Social Care teams, carers and AAC PLWD. For LA social care providers and practitioners, we will produce:

- broadcast quality podcasts, audiobooks, short films, and downloadable documents
- training material for staff focused on how to respond, recognise, and intervene to support AAC PLWD
- recommendations delivering guidance on recognising the support needs of AAC PLWD, identifying opportunities to provide support, and promoting good practices.

For AAC PLWD, their care partners, and families, we will produce:

- culturally appropriate guides on accessing services, support, and the risks of crisis
- short accessible factsheets on living with dementia and rights to reasonable adjustments.

All materials will be freely accessible to the public in multiple formats and languages including French, Arabic, Hausa, Igbor, Swahili, and Somali.

Our strategy will build on the team’s prior work to ensure wide reach and engagement. This will be done through:

- engagement with BASW, ADASS and the National association of Principal Social workers forum to disseminate findings
- AAC PLWD, their care partners, and families through a programme of public outreach PPI events and consultations
- participating Adult Social Care teams and sites, to build understandings and recognition of pathways to and access to services, for exploring key social processes and institutional forces at play in how support needs are understood, become recognised, and responded to, the impacts and the potential for alternative approaches
- The PAG, to share and discuss emerging definitions of what constitutes the provision culturally appropriate care and making reasonable adjustments to advise on the appropriate boundaries of training and interventions to support social care staff and the interface with people living with dementia and families. Members include service users, third sector organisations and networks, experts in dementia care, medical ethics and law.

### Status and timeline

The only stage that has been completed at present is receiving all required ethical approvals; all other stages have not been completed. Participant recruitment for the ethnographic observations, narrative interviews and artisitic dialogue and workshops has started. Below is the study timeline, with a status provided for each key milestone:

- *June 2025* – receive ethics approvals by (status: completed)
- *June 2027 –* complete participant recruitment by (status: underway; not complete)
- *June 2027* – complete data collection by (status: commenced; not complete)
- *June 2027* – complete analysis by (status: not complete)
- *December 2027* – finalise results and final report by; conduct dissimination by (status: not complete)

## Discussion and limitations

A strength of this study is that it will be the first to specifically focus on older PLWD within AAC communities using an intersectional theoretical framework. The ethnographic aspects of this study will also provide an empirical foundation for future research and interventions.

In terms of limitations, we acknowledge the Hawthorne effect, whereby a person modifies their behaviour while under observation. The signicant number of days of observation should both minimise this and provide accurate data of how decisions are made in everyday practice. We are also aware that demographic diversity in other regions may produce findings that differ from those that will be observed in this study; this will be explicitly acknowledged in our discussion of the results and the development of recommendations.
